# Rosiglitazone as an option for patients with acromegaly: a case series

**DOI:** 10.1186/1752-1947-5-200

**Published:** 2011-05-21

**Authors:** Héctor E Tamez-Pérez, Ana Bahena-García, María D Gómez de Ossio, Hugo Gutiérrez-Hermosillo, Alejandra L Tamez-Peña

**Affiliations:** 1 Subdirección de Investigación, Facultad de Medicina, Universidad Autónoma de Nuevo León, Monterrey, N.L., México; 2 Av. Francisco I. Madero Pte. s/n y Dr. Aguirre Pequeño, Mitras Centro, C.P. 64460 Monterrey, N.L., México; 3 Hospital de Especialidades #25 IMSS, Monterrey, N.L., México; 4 Lincoln y Gonzalitos s/n. Col. Nueva Morelos. Monterrey, N.L., México

## Abstract

**Introduction:**

In the patient with acromegaly, pituitary surgery is the therapeutic standard. Despite undergoing surgery, a significant number of patients with acromegaly continue to have uncontrolled growth hormone secretion. These patients require other treatments such as external irradiation and/or drug therapy.

**Case presentation:**

We present the clinical and laboratory responses to six months of treatment with rosiglitazone in four cases. In all four cases, the patients had persistent growth hormone overproduction despite previous surgical treatment and other conventional therapy. Case 1 is a 57-year-old Caucasian woman, case 2 is a 51-year-old Hispanic man, case 3 is a 32-year-old Hispanic woman, and case 4 is a 36-year-old Hispanic man. In three of these patients, basal and nadir growth hormone and insulin-like growth factor 1 levels were significantly decreased (*P *< 0.05 and *P *< 0.01, respectively).

**Conclusion:**

Rosiglitazone could be a treatment option in select patients with acromegaly.

## Introduction

Pituitary tumors represent 10% to 15% of brain tumors, and their presentation is characterized by clinical data that can be summarized in three aspects: endocrine, with hypopituitarism or excessive secretion of pituitary hormone; a mass effect; and diverse neurological alterations [1].

The standard treatment of small and non-functional tumors is surveillance, but in the patient with acromegaly, pituitary surgery is the therapeutic standard. With surgery, cure and/or remission depend on several factors, such as tumor size and extension, degree of infiltration, and the surgical team's experience. Recently published guidelines for the goals of treatment as well as the appropriate imaging, biochemical, and clinical monitoring of patients with acromegaly have been enunciated, based on the available published evidence [2].

Despite undergoing surgery, a significant number of patients with acromegaly continue to have uncontrolled growth hormone (GH) secretion. These patients require other treatments, such as external irradiation and/or drug therapy [2,3].

Even multimodal therapy fails to control tumor progression in some patients, which is why other treatment methods have been explored. There are two previous reports of patients with acromegaly treated with rosiglitazone, a peroxisome proliferator-activated receptor γ (PPAR-γ) agonist [4,5], as well as two recent *in vitro *studies [6,7].

The aim of the present case series is to present our experience with rosiglitazone as rescue therapy in four patients with acromegaly who had active disease. This case series was approved by the local ethics committee.

## Case presentation

Case 1 is a 57-year-old Caucasian woman diagnosed twelve years ago with acromegaly on the basis of clinical, radiological, and laboratory tests. Her first treatment was transcranial surgery. Because of biochemical data showing disease activity, she underwent additional treatment with radiotherapy and octreotide for more than two years. An evaluation of blood samples based on assays for glucose, insulin-like growth factor 1 (IGF-1), and growth hormone (GH) performed 0, 30, 60, 90, and 120 minutes after a 75 g oral glucose load showed an absence of normalization of hormone levels in clinical evaluation and led to the initiation of treatment with rosiglitazone 4 mg orally as a single morning dose for a period of six months. Clinical and biochemical evaluations performed at three and six months showed statistically significant differences with regard to GH and IGF-1 levels (*P *< 0.05 and *P *< 0.001, respectively). Treatment with rosiglitazone was well-tolerated, and there were no changes in standard biochemical profiles.

Case 2 is a 51-year-old Hispanic man with a diagnosis of acromegaly since 2002. His initial treatment was transcranial surgery, and because he had laboratory abnormalities without radiological evidence of a tumor, he received octreotide therapy for two years. An evaluation of blood samples based on assays for glucose, IGF-1, and GH performed 0, 30, 60, 90, and 120 minutes after a 75 g oral glucose load showed an absence of normalization of hormone levels in clinical evaluation, which led us to initiate treatment with rosiglitazone 4 mg orally as a single morning dose for a period of six months. This treatment failed to achieve normalization of GH and IGF-1 levels (*P *> 0.05). Treatment with rosiglitazone was well-tolerated, and there were no changes in standard biochemical profiles.

Case 3 is a 32-year-old Hispanic woman diagnosed with acromegaly ten years ago on the basis of clinical, radiological, and laboratory results. Her first treatment was transsphenoidal surgery, without improvement in clinical or laboratory values. She received radiotherapy and octreotide treatment three years ago. An evaluation of blood samples based on assays for glucose, IGF-1, and GH performed 0, 30, 60, 90, and 120 minutes after a 75 g oral glucose load showed an absence of normalization of hormone levels based on clinical evaluation, and she was treated with rosiglitazone 4 mg orally in a single morning dose for a period of six months. Her clinical and biochemical evaluations at three and six months showed statistically significant differences in GH and IGF-1 levels (*P *< 0.05 and *P *< 0.001, respectively). Treatment with rosiglitazone was well-tolerated, and there were no changes in standard biochemical profiles.

Case 4 is a 36-year-old Hispanic man who was diagnosed with acromegaly seven years ago on the basis of clinical, radiological, and biochemical laboratory results. His initial treatment was transesphenoidal surgery. Because of active disease without tumor as determined by imaging tests, he received octreotide for more than three years. An evaluation of based on blood samples for glucose, IGF-1, and GH assays performed at 0, 30, 60, 90, and 120 minutes after a 75 gram oral glucose load showed an absence of normalization of hormone levels based on clinical evaluation, and we initiated treatment with rosiglitazone 4 mg orally as a single morning dose for a period of six months. Post-treatment evaluation showed significantly decreased levels of GH and IGF-1 (*P *< 0.05 and *P *< 0.001, respectively). Treatment with rosiglitazone was well-tolerated, and there were no changes in standard biochemical profiles.

## Discussion

The demographic information and treatment results of our four patients are shown in Table 1. In at least 95% of cases, acromegaly is caused by a GH-secreting pituitary adenoma. In view of the increased mortality associated with acromegaly, successful management is of the utmost importance, especially because reduction of GH concentration produces a decrease in mortality [1-3].

**Table 1:** Patient demographics and laboratory findings^a^

								3 months		6 months	
								
Patient	Sex	Age, years	Previous treatment	GH normal range, ng/ml	Basal GH level, ng/ml	Nadir GH level, ng/ml	IGF-1 level, ng/ml (normal value for age)	GH level, ng/ml	IGF-1 level, ng/ml	GH level, ng/ml	IGF-1 level, ng/ml
1	Woman	57	S, R, O	0.003 to 0.97	10.0	4.32	990 (80 to 197)	3.26	400	1.24	284
2	Man	51	S, O	0.010 to 3.60	12	4.01	1016 (80 to 157)	20.73	1700	9.39	540
3	Woman	32	S, R, O	0.003 to 0.97	47	29.9	1640 (98 to 226)	11.1	715	4.29	635
4	Man	36	S, O	0.010 to 3.60	84	71	2400 (98 to 226)	51	2850	11	375

^**a**^S: surgery, R: radiotherapy, O: octreotide lar.

Transsphenoidal surgery is currently the first-line treatment for acromegaly. Remission is observed in 80% to 90% of patients with microadenomas, in 50% to 60% of patients with non-invasive macroadenomas, and in fewer than 20% of patients with invasive macroadenomas [8].

The low success rate of surgical treatment has led to an increased interest in medical therapies for patients with acromegaly. Three types of drugs are available: somatostatin analogues for primary or adjunctive therapy, which adequately control GH and IGF-1 in 60% to 75% of patients; dopamine agonists, which are associated with a control rate that is unacceptably low; and pegvisomant, which normalizes IGF-1 levels in more than 90% of patients. Despite the use of combined therapy (surgery and other treatments, including drugs and radiotherapy), a significant number of patients with acromegaly continue to have active disease. The factors associated with tumor activity are patient characteristics, tumor morphology, and genetic markers [1-3,8].

The molecular biology of these tumors has been investigated recently, with findings of over-expression of PPAR-γ in pituitary tissue as in other types of neoplasia, such as breast, colon, or prostate cancer [6-11].

The activation of PPAR-γ receptors inhibits tumor growth in rodents and induces the oncostatic effect in human cancer cell lines. In one study [6], there was no correlation between PPAR-γ expression and the efficacy of rosiglitazone, suggesting that the action of this compound is independent of PPAR-γ expression and thus may be useful in the treatment of non-functioning pituitary adenomas. However, its efficacy in patients with Cushing's disease and acromegaly requires further study.

A beneficial effect of rosiglitazone has recently been demonstrated in *in vitro *studies, animal models, and short- and long-term clinical studies in patients with central Cushing's syndrome [12]. Rosiglitazone has been a widely accepted drug for the treatment of type 2 diabetes mellitus since 1999 [13].

A previous report described one case of active acromegaly and type 2 diabetes mellitus in which a tumor was not completely removed and octreotide, a somatostatin analogue, was introduced but rapidly discontinued one week afterward because of intolerance. Rosiglitazone was introduced as an alternative treatment for hyperglycemia, and after 90 days of therapy with the drug, GH and IGF-1 levels decreased and caused amelioration of acromegaly features [4].

In another report, four patients did not demonstrate a significant response with regard to GH and IGF-1 levels before and after treatment with an 8 mg oral dose of rosiglitazone [5].

Three of our four patients presented decreased GH and IGF-1 levels after being treated with only 4 mg rosiglitazone (the usual dose for hyperglycemia in México) for six months. Despite other reports describing the use of higher doses, we suggest that dose variation, treatment time, and maybe genetic factors are the causes of the different results described previously.

It is also important to consider that treatment of acromegaly is effective in reducing morbidity and mortality when biochemical profiles are below "safe" hormone levels, which were not reached in any of the cases [1,2].

## Conclusion

In selected patients with acromegaly, rosiglitazone therapy may decrease GH and IGF-1 levels. Further research with rosiglitazone, alone or in combination with other treatment modalities, is required to define its possible role in long-term medical therapy.

## Abbreviations

GH: growth hormone; IGF-1: insulin-like growth factor 1; PPAR-γ: peroxisome proliferator-activated receptor γ.

## Competing interests

The authors declare that they have no competing interests.

## Consent

Written informed consent was obtained from the patients for publication of this case series and any accompanying images. A copy of the written consent is available for review by the Editor-in-Chief of this journal.

## Authors' contributions

TH analyzed and interpreted the patient data and was a major contributor in writing the manuscript. Both BA and GM analyzed and interpreted the patient data. GH analyzed and interpreted laboratory studies. TA analyzed and interpreted the laboratory studies and the statistical reporting. All authors read and approved the final manuscript.
